# dCubilin- or dAMN-mediated protein reabsorption in *Drosophila* nephrocytes modulates longevity

**DOI:** 10.1242/dmm.047464

**Published:** 2021-09-21

**Authors:** Xiaoming Feng, Xizhen Hong, Qiuxia Fan, Liting Chen, Jing Li, Juan Deng, Siqiao Gong, Fan Fan Hou, Fujian Zhang

**Affiliations:** 1 State Key Laboratory of Organ Failure Research, National Clinical Research Center of Kidney Diseases, Division of Nephrology, Nanfang Hospital, Southern Medical University, Guangzhou 510515, China; 2 Bioland Laboratory (Guangzhou Regenerative Medicine and Health Guangdong Laboratory), Guangzhou 510320, China; 3 North China University of Science and Technology Affiliated Hospital, Tangshan, Hebei 063000, China; 4 The Eighth Affiliated Hospital, Sun Yat-sen University, Shenzhen 518033, China

**Keywords:** *Drosophila*, Nephrocytes, dCubilin- and dAMN-mediated protein reabsorption, Aging, Muscle senescence, Neurodegeneration, Tele-proteostasis

## Abstract

Aging is a multifaceted process regulated by multiple cellular pathways, including the proteostasis network. Pharmacological or genetic enhancement of the intracellular proteostasis network extends lifespan and prevents age-related diseases. However, how proteostasis is regulated in different tissues throughout the aging process remains unclear. Here, we show that *Drosophila* homologs of Cubilin- and Amnionless (dCubilin and dAMN, respectively)-mediated protein reabsorption (CAMPR) from hemolymph insect blood by nephrocytes modulate longevity through regulating proteostasis in muscle and brain tissues. We find that overexpression of dAMN receptor in nephrocytes extends lifespan, whereas nephrocyte-specific *dCubilin* or *dAMN* RNAi knockdown shortens lifespan. We also show that CAMPR in nephrocytes regulates proteostasis in hemolymph and improves healthspan. In addition, we show that enhanced CAMPR in nephrocytes slows down the aging process in muscle and brain by maintaining the proteostasis network in these tissues. Altogether, our work has revealed an inter-organ communication network across nephrocytes and muscle/neuronal tissue that is essential for maintaining proteostasis, and to delay senescence in these organs. These findings provide insight into the role of renal protein reabsorption in the aging process via this tele-proteostasis network.

## Abstract (precis)

**Summary:** dCubilin- or dAMN-mediated protein reabsorption in nephrocytes from hemolymph modulates *Drosophila* lifespan through regulating proteostasis and aging in muscle and brain tissues via the tele-proteostasis mechanism.

## INTRODUCTION

Aging is an immensely complex process regulated by multiple interacting cellular pathways. Not surprisingly, numerous lines of evidence support a tight relationship between proteostasis and healthy aging (Kaushik and Cuervo, 2015; Klaips et al., 2018; Korovila et al., 2017; Labbadia and Morimoto, 2015; Santra et al., 2019; Taylor and Dillin, 2011). The proteostasis network consists of molecular chaperones, stress-response transcription factors and protein degradation machines that sense and respond to proteotoxic stress, as well as protein misfolding, to ensure cell viability (Kaushik and Cuervo, 2015). Chaperones and two proteolytic systems, the ubiquitin proteasome system and the lysosome-autophagy system, take charge of the maintenance of intracellular proteostasis. It has been shown that the activity of these systems dramatically decreases with aging (Cuervo and Wong, 2014; Revuelta and Matheu, 2017; Shirakabe et al., 2016; Wong et al., 2020). Enhancing proteasome or autophagy activity by overexpressing proteasome subunits or essential autophagy genes has resulted in extended lifespan in model organisms, such as *Saccharomyces cerevisiae*, *Caenorhabditis elegans* and *Drosophila melanogaster* (Cheon et al., 2019; Chondrogianni et al., 2015; Madeo et al., 2015; Pyo et al., 2013). To date, most studies on age-related changes in proteostasis consider it to be a cell-autonomous process. However, the existence of intercellular or inter-organ proteostasis networks (tele-proteostasis) that help coordinate the response of tissues and organs to proteotoxic insults has been proposed (Kaushik and Cuervo, 2015). The observation of tele-proteostasis, such as the integration of distant networks, and its potential implications for identifying novel regulatory mechanisms and functional attributes for proteostasis are compelling. However, additional evidence of such tele-proteostasis is lacking.

Interestingly, results from a series of studies support the notion that the plasma proteome harbors key regulators of aging. Using a heterochronic parabiosis method that connects the circulatory systems of young and old mice, these studies showed that multiple tissues, including heart, kidney, muscle, brain, liver, bone and pancreas, can be rejuvenated in old mice (Conboy et al., 2013; Conese et al., 2017; Eggel and Wyss-Coray, 2014; Smith et al., 2015). Plasma from old mice is sufficient to accelerate brain aging after infusion into young mice, whereas young plasma is able to reverse these aspects of brain aging (Smith et al., 2015). Since these initial findings, plasma proteomic changes with aging have been thoroughly exploited, and changes in protein expression across the lifespan have been linked to biological pathways and diseases (Lehallier et al., 2019). However, how these plasma proteomic changes during the aging process are regulated remains unclear.

*Drosophila* has been widely used to study the aging process because of its short lifespan and easy genetic manipulation. The *Drosophila* excretory system is composed of nephrocytes (which regulate hemolymph composition by filtration followed by filtrate endocytosis) and Malpighian tubules (which modify and secrete urine). It has been shown that the *Drosophila* nephrocyte shares remarkable similarity with the glomerular podocyte for protein ultrafiltration, and the renal proximal tubule for protein reabsorption (Na and Cagan, 2013; Weavers et al., 2009; Zhang et al., 2013a). *Drosophila* nephrocytes can be divided into two distinct groups: the garland cells, which appear as a necklace-like structure surrounding the esophagus, and the *Drosophila* pericardial cells that form two rows of cells flanking the heart (Cagan, 2011; Narita et al., 1989). In the adult stage, pericardial nephrocytes serve as the primary filtration units. In our previous studies, we showed that *Drosophila* homologs of mammalian cubilin and amnionless (AMN), two major receptors for protein reabsorption in renal proximal tubules, are required for nephrocyte protein reabsorption from the hemolymph *in vivo*. We also showed that dCubilin- or dAMN-mediated protein reabsorption is essential for toxin removal (Zhang et al., 2013a). It has been shown that *Drosophila* nephrocytes remove microbiota-derived peptidoglycan from the hemolymph to maintain immune homeostasis (Guillou et al., 2016). These findings indicate that nephrocytes are important for proteostasis; however, gaps exist in our knowledge of the exact contribution of nephrocytes, and their potential role in tele-proteostasis remains unknown.

*Drosophila* pericardial nephrocyte ultrastructure changes during aging (Psathaki et al., 2018). However, it is still not clear whether pericardial nephrocytes could also regulate lifespan. In this study, we showed that dCubilin- or dAMN-mediated protein reabsorption from hemolymph by *Drosophila* nephrocytes modulates longevity by regulating proteostasis in muscle and brain tissues, providing evidence of a tele-proteostasis network. To evaluate the role of protein reabsorption in *Drosophila* nephrocytes in the aging process, we manipulated the expression of AMN receptor protein specifically in nephrocytes. Our results showed that enhanced protein reabsorption in nephrocytes extended *Drosophila* lifespan, whereas decreased protein reabsorption resulted in shorter lifespans. Further, we showed that dCubilin- or dAMN-mediated protein reabsorption in nephrocytes regulates proteostasis in hemolymph and improves *Drosophila* healthspan. To explore the molecular mechanism through which dCubilin- and dAMN-mediated protein reabsorption in nephrocytes regulates lifespan, we examined its effect on long-distance organs, such as brain and muscle. We found that enhanced dCubilin- or dAMN-mediated protein reabsorption in nephrocytes slows down the aging process in muscle and brain by maintaining the proteostasis network. Therefore, our study provides evidence for the existence of a tele-proteostasis network that coordinates proteostasis across multiple organs in *Drosophila*. Together, these findings suggest that renal protein reabsorption may play a major role in the regulation of the plasma proteomic changes during aging.

## RESULTS

### Increased protein accumulation in *Drosophila* hemolymph with aging

**Fig. 1.**
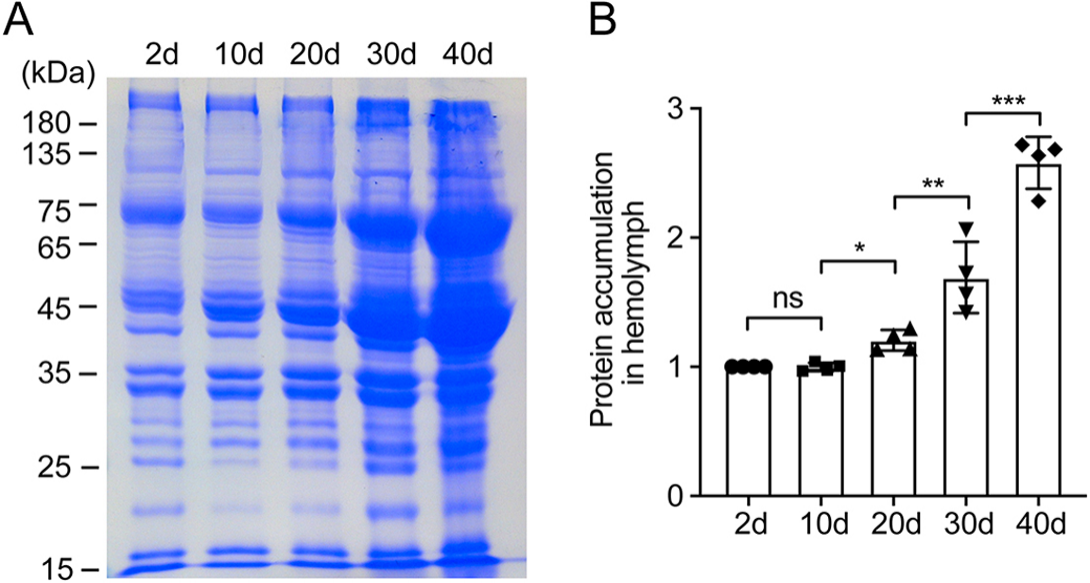
**Increased protein accumulation in *Drosophila* hemolymph with aging.** (A) SDS-PAGE analysis of the *Drosophila* hemolymph proteins extracted from 2-day-, 10-day-, 20-day-, 30-day- and 40-day-old control flies. Protein accumulation increased in *Drosophila* hemolymph with aging. (B) Quantitative data show that *Drosophila* hemolymph protein accumulation significantly increased with aging. Data are mean±s.d. and representative of four independent experiments. *P<0.05; **P<0.01; ***P<0.001; ns, not significant (one-way ANOVA).

To test whether protein content in the plasma increases with aging, we examined protein accumulation in hemolymph extracts from 2-day-, 10-day-, 20-day-, 30-day- and 40-day-old flies (20 males and 20 females). As the hemolymph clots very quickly upon injury, 2× SDS loading buffer was added in the collecting tubule, which will intervene with the Bradford protein analysis. Thus, SDS-PAGE analysis was used to assess total protein content in hemolymph. The total volume of hemolymph extracted from 40 flies was very small (less than 5 µl) and there are no standard internal markers for hemolymph; therefore, all the extracts were loaded onto SDS-PAGE to eliminate the technical artifact in our experiments. As shown in Fig. 1A, the amount of total proteins in hemolymph significantly increased with aging. The 40-day-old flies showed a more than twofold increase in the amount of total proteins in hemolymph compared to 2-day-old flies (Fig. 1B). Our results suggest that protein accumulation in hemolymph could be used as an aging marker in *Drosophila*.

### Knockdown of dAMN or dCubilin in nephrocytes exaggerates protein accumulation in *Drosophila* hemolymph

**Fig. 2.**
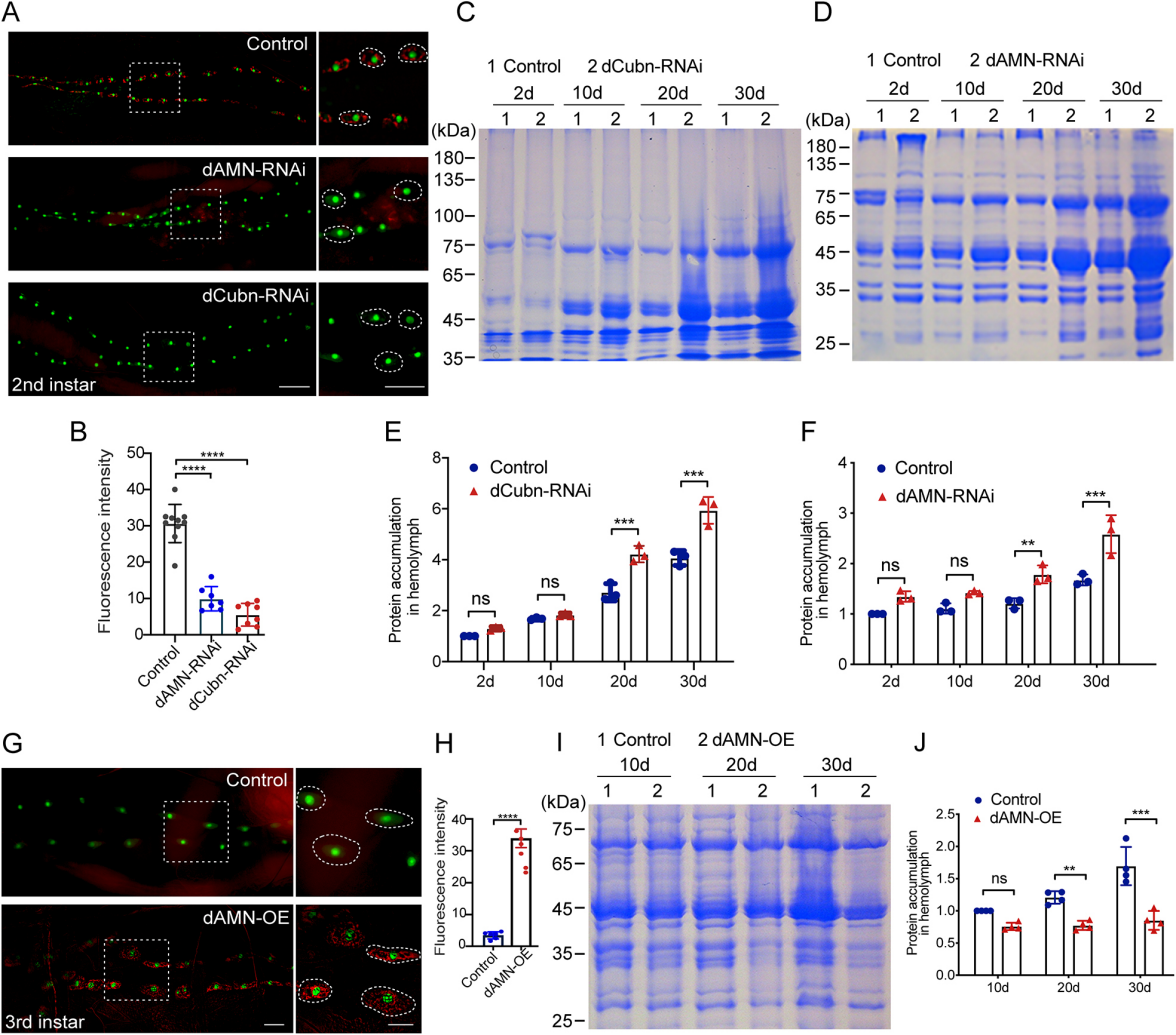
**Protein accumulation with aging in *Drosophila* hemolymph was regulated by dCubilin- or dAMN- mediated protein reabsorption in nephrocytes.** Representative micrographs of nephrocytes of second instar larvae. Secreted ANF-RFP (red) is accumulated in pericardial nephrocytes labeled with Hand-GFP (green). Knockdown of *dAMN* or *dCubilin* in nephrocytes led to decreased protein reabsorption in nephrocytes (right panels, higher magnified views of the *Drosophila* nephrocytes). (B) Quantitative data show that *dCubilin* or *dAMN* RNAi knockdown resulted in decreased protein reabsorption. (C,D) SDS-PAGE analysis of the *Drosophila* hemolymph proteins extracted from 2-day-, 10-day-, 20-day- and 30-day-old control and *dCubilin* (C) and *dAMN* (D) knockdown flies. (E,F) Quantification of SDS-PAGE analysis of hemolymph proteins from *dCubilin* (E) or *dAMN* (F) knockdown flies. (G) Representative micrographs of nephrocytes of third instar larvae. Little ANF-RFP (red) was observed in pericardial nephrocytes in the control, whereas overexpression of dAMN in pericardial nephrocytes led to the accumulation of ANF-RFP (right panels, higher magnified views of the *Drosophila* nephrocytes). (H) Quantitative data show that dAMN overexpression resulted in increased protein reabsorption. (I) SDS-PAGE analysis of the *Drosophila* hemolymph proteins extracted from 10-day-, 20-day- and 30-day-old control and dAMN overexpression flies. (J) Quantification of SDS-PAGE analysis of hemolymph proteins from control and dAMN overexpression flies. Data in B and H were acquired from at least six individual replicates. Data in E, F and J were representative of at least three independent experiments. Data are mean±s.d. **P<0.01; ***P<0.001; ****P<0.0001; ns, not significant [unpaired and two-tailed Student's *t*-test (B); Mann–Whitney U non-parametric test (E,F,J); one-way ANOVA (H)]. Scale bars: 50 μm.

Total protein in hemolymph increases with age, perhaps because nephrocytes have decreased reabsorption or tissues release more proteins into the hemolymph. Additionally, the increase could occur as a result of gut leakage. dCubilin and dAMN are two receptors that are essential for protein reabsorption in *Drosophila* nephrocytes from the hemolymph, as well as in renal proximal tubular cells of mammals from the crude urine. To explore the role of protein reabsorption in nephrocytes in maintaining hemolymph protein hemostasis, we knocked down dCubilin or dAMN genes specifically in nephrocytes using the Dot-Gal4/UAS-RNAi system and examined the effect on hemolymph protein accumulation. PH,pMAR; Dot-Gal4 virgins were crossed with UAS-dAMN-RNAi and UAS-dCubilin-RNAi male flies to specifically knockdown the dAMN or dCubilin genes in nephrocytes. The progenies of PH,pMAR; Dot-Gal4 virgins crossed with W^1118^ male flies were used as control in all the following experiments. As shown in Fig. 2A,B, RNAi knockdown of dAMN or dCubilin in nephrocytes resulted in decreased protein reabsorption in nephrocytes of second instar larvae. Compared to the control group, RNAi knockdown of *Drosophila* dCubilin in nephrocytes also resulted in increased protein accumulation in hemolymph in 20-day- and 30-day-old flies (Fig. 2C,E). Similarly, RNAi knockdown of dAMN in nephrocytes led to increased protein accumulation in hemolymph in 20-day- and 30-day-old flies (20 males and 20 females per group; Fig. 2D,F).

### Overexpression of dAMN in nephrocytes ameliorates protein accumulation in *Drosophila* hemolymph

Next, we investigated whether enhancing protein reabsorption in nephrocytes ameliorates protein accumulation in *Drosophila* hemolymph. We overexpressed dAMN specifically in nephrocytes and examined its effect on protein reabsorption and hemolymph protein accumulation. Hemolymph proteins were extracted from controls or flies overexpressing dAMN protein at different age groups, and were then subjected to SDS-PAGE analysis. As shown in Fig. 2G,H, overexpression of dAMN in nephrocytes using the Dot-Gal4 driver led to increased red fluorescent protein (RFP) reabsorption in third instar nephrocytes. Compared to the control group, overexpression of dAMN in nephrocytes also resulted in decreased hemolymph protein accumulation in 20-day- and 30-day-old flies (Fig. 2I,J).

### Decreased protein reabsorption in nephrocytes shortens *Drosophila* lifespan

**Fig. 3.**
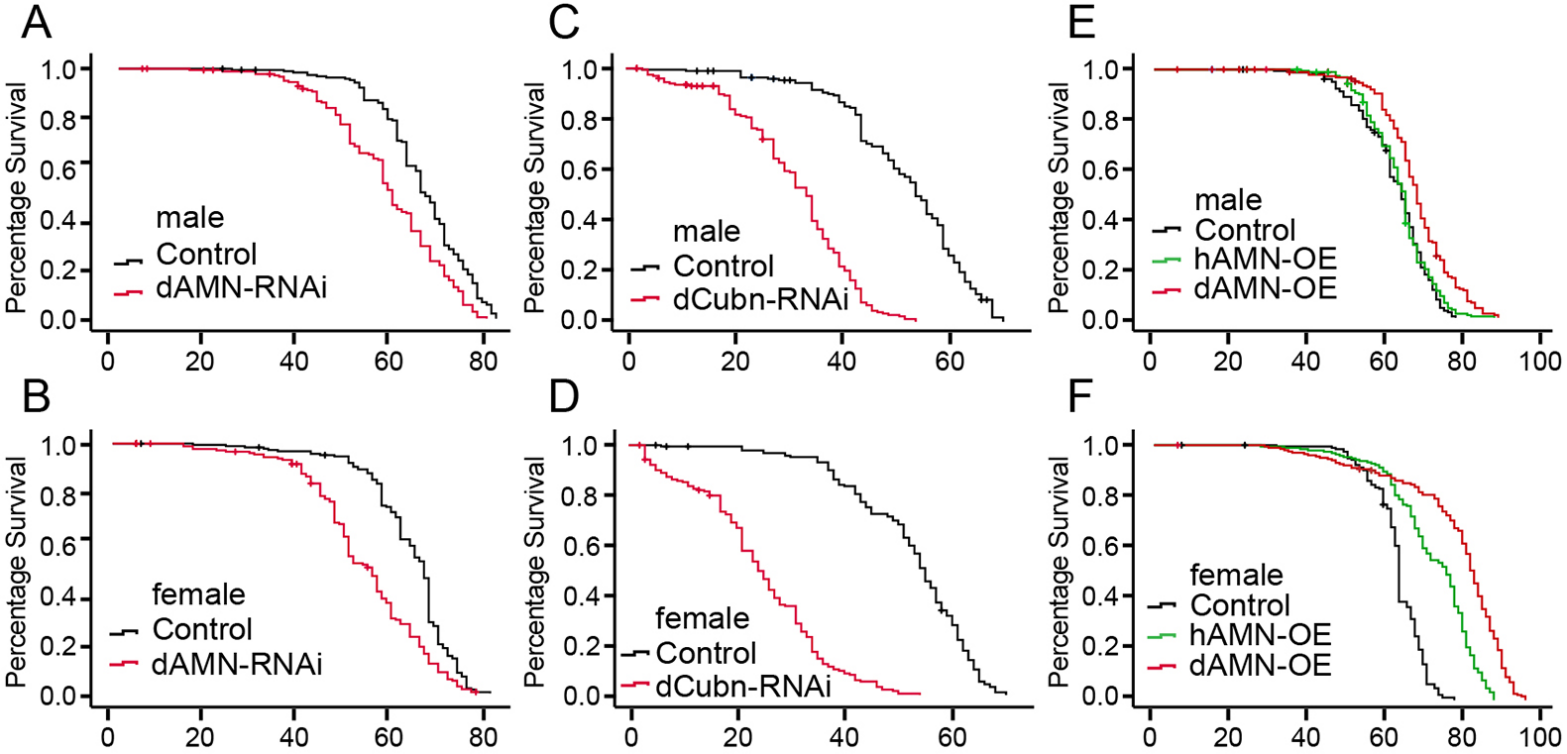
***dAMN* or *dCubilin* knockdown in nephrocytes shortens *Drosophila* lifespan, whereas dAMN overexpression in nephrocytes extends *Drosophila* lifespan.** (A-F) Survival curves of flies with different genotypes. The data of each genotype was acquired from at least 190 individual replicates. The log rank test was used for significance testing. (A,B) RNAi knockdown of *dAMN* specifically in nephrocytes significantly shortened lifespan in male and female flies (average 57.1 days versus 64.6 days for male; average 53.5 days versus 63.1 days for female; *P*<0.05). (C,D) RNAi knockdown of *dCubilin* specifically in nephrocytes dramatically shortened lifespan in male and female flies (average 31 days versus 51.4 days for male; average 24.4 days versus 52.4 days for female; *P*<0.05). (E,F) Nephrocyte-specific dAMN overexpression significantly extended lifespan in male and female flies (average 67.9 days versus 62.4 days for male; average 76.7 days versus 61.9 days for female; *P*<0.05). Nephrocyte-specific overexpression of human AMN (hAMN) significantly extended lifespan in female flies (average 70.8 days versus 61.9 days, *P*<0.05).

To investigate whether protein reabsorption in nephrocytes affects *Drosophila* lifespan, we knocked down *dCubilin* or *dAMN* genes specifically in nephrocytes using the Dot-Gal4/UAS-RNAi system and examined the effect on *Drosophila* lifespan. As shown in Fig. 3A-D, RNAi knockdown of dAMN or dCubilin in nephrocytes significantly shortened the lifespan of both female and male flies, supporting a role for dCubilin- or dAMN-mediated protein reabsorption in nephrocytes in sustaining lifespan.

### Increased protein reabsorption in nephrocytes extends *Drosophila* lifespan

To test whether enhancing protein reabsorption in nephrocytes extends lifespan, we overexpressed *Drosophila* and human AMN proteins (dAMN and hAMN, respectively) specifically in nephrocytes and examined their effect on lifespan. As shown in Fig. 3E,F, overexpression of dAMN, but not hAMN, in nephrocytes led to significantly extended lifespan in male flies compared with the control group (62.4 days for the control group versus 67.9 days for the dAMN overexpression group). Overexpression of dAMN or hAMN in nephrocytes significantly extended the lifespan of female flies (61.9 days for the control group versus 76.9 days for dAMN and 70.8 days for hAMN).

### Defect in protein reabsorption in nephrocytes impairs *Drosophila* healthspan

**Fig. 4.**
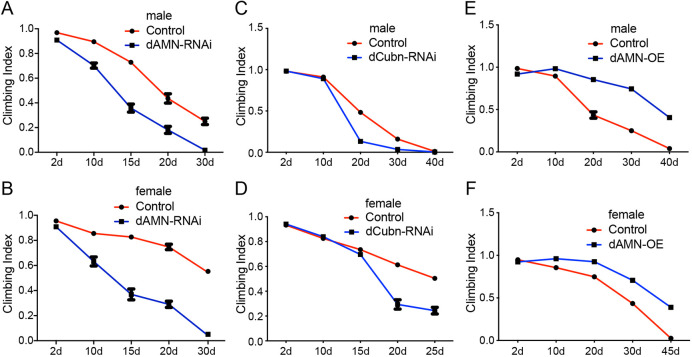
***dAMN* or *dCubilin* knockdown in nephrocytes impairs climbing ability, whereas dAMN overexpression in nephrocytes improves climbing ability*.*** (A,B) The climbing ability of *dAMN* RNAi knockdown flies dramatically decreased compared to the control group (CI_50_=13.8 days versus CI_50_=31 days for females, *P*<0.001; CI_50_=13.4 days versus CI_50_=21 days for males, *P*<0.001). (C,D). The climbing ability of *dCubilin* RNAi knockdown flies dramatically decreased compared to the control group (CI_50_=17.4 days versus CI_50_=25 days for females, *P*<0.001; CI_50_=17.3 days versus CI_50_=20.7 days for males, *P*<0.001). (E,F). The climbing ability of dAMN overexpression flies significantly improved compared to controls (CI_50_=37 days versus CI_50_=26 days for females, *P*<0.001; CI_50_=30 days versus CI_50_=20 days for males, *P*<0.001). Data were acquired from ten replicate groups. Values are expressed as mean±s.d. The CI was calculated by dividing the number of flies passing the 8-cm line mark by total fly numbers. CI_50_ represents the age of flies with 50% of flies passing the 8-cm line mark.

The negative geotaxis assay, the natural tendency of flies to move against gravity when agitated, has been widely used to study genes or conditions that may hinder locomotor capacity. To test whether protein reabsorption in nephrocytes affects *Drosophila* healthspan, we knocked down dCubilin or dAMN proteins specifically in nephrocytes using the Dot-Gal4/UAS-RNAi system and examined their effect on locomotor ability. As shown in Fig. 4, dAMN or dCubilin knockdown in nephrocytes resulted in decreased climbing ability in both male and female flies (Fig. 4A-D). Compared to control male flies, male flies with dAMN knockdown exhibited significantly decreased climbing ability [climbing index (CI)_50_=13.4 days versus CI_50_=21 days (control), *P*<0.001; Fig. 4A]. The climbing ability of female flies with the dAMN knockdown dramatically decreased compared to the control group [CI_50_=13.8 days versus CI_50_=31 days (control), *P*<0.001; Fig. 4B]. RNAi knockdown of dCubilin led to a similar result, with CI_50_=17.3 days versus CI_50_=20.7 days (control) (*P*<0.001) for males (Fig. 4C), and CI_50_=17.4 days versus CI_50_=25 days (control) (*P*<0.001) for females (Fig. 4D). These findings reveal that defects in protein reabsorption in nephrocytes impair *Drosophila* healthspan.

### Enhancing protein reabsorption in nephrocytes improves *Drosophila* healthspan

To examine whether protein reabsorption in nephrocytes affects *Drosophila* locomotor capacity, we overexpressed dAMN specifically in nephrocytes and examined its effect on healthspan as measured by geotaxis. As shown in Fig. 4E,F, dAMN overexpression in nephrocytes led to increased climbing ability in both male and female flies. Compared to control male flies, male flies overexpressing dAMN significantly improved climbing ability [CI_50_=30 days versus CI_50_=20 days (control), *P*<0.001; Fig. 4E]. Female flies with dAMN overexpression also showed significantly improved climbing ability [CI_50_=37 days versus CI_50_=26 days (control), *P*<0.001; Fig. 4F]. These results strongly indicate that enhanced protein reabsorption in nephrocytes improves *Drosophila* healthspan.

### dCubilin- or dAMN-mediated protein reabsorption in nephrocytes affects *Drosophila* brain proteostasis and aging

**Fig. 5.**
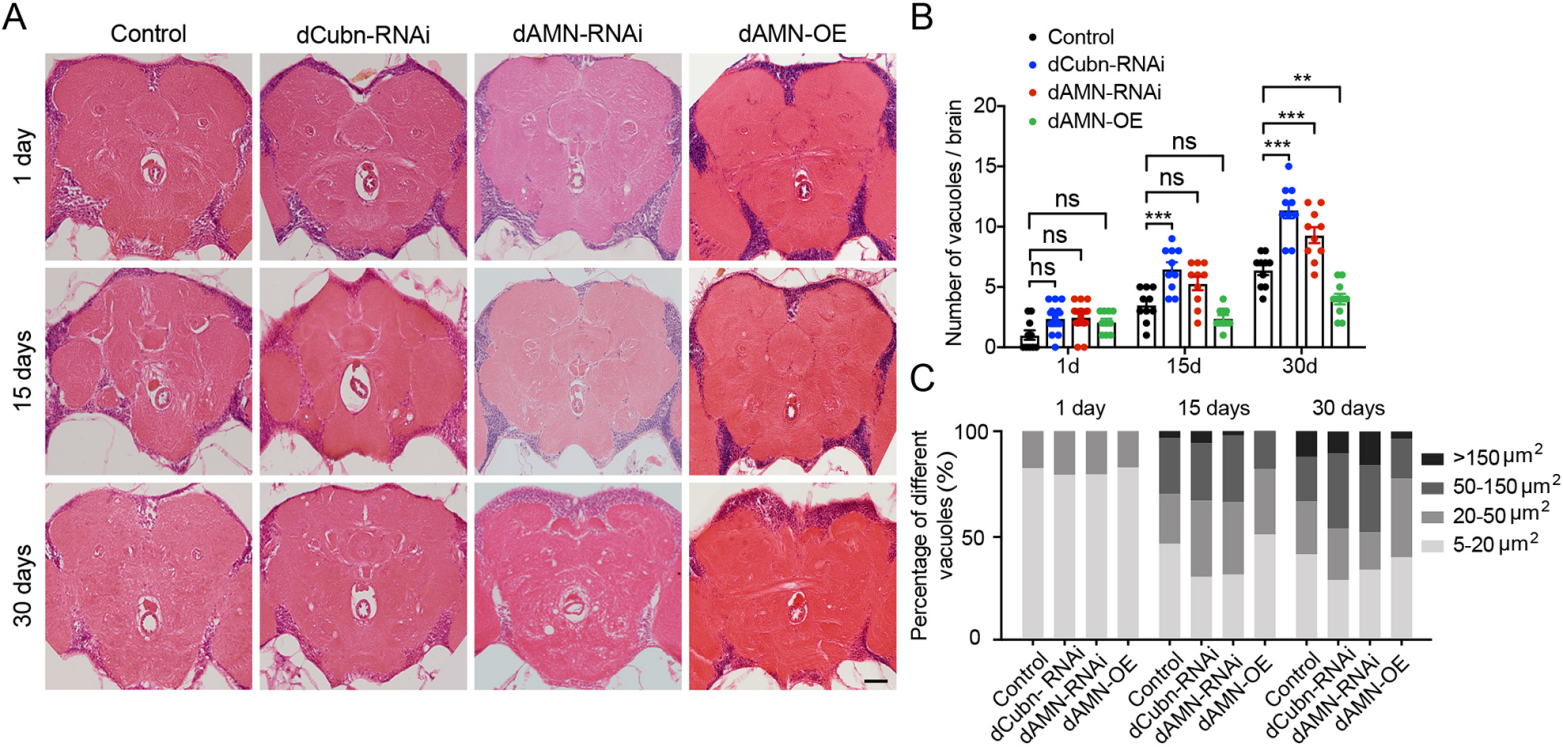
**dCubilin- or dAMN-mediated protein reabsorption in nephrocytes affects the formation of vacuoles in *Drosophila* brain.** (A) Representative micrographs of vacuoles in brains of flies with different genotypes. Scale bar: 50 μm. (B) Quantification of the number of vacuoles in brains of 1-day-, 15-day- and 30-day-old flies with different genotypes, as indicated (*n*=10). **P<0.01; ***P<0.001; ns, not significant (one-way ANOVA). Data are mean±s.d. (C) The percentage of each size range of vacuoles in brain tissue of 1-day-, 15-day- and 30-day-old flies with different genotypes, as indicated.

The formation of vacuoles in *Drosophila* brain was previously linked to oxidative damage and accelerated aging (Cabirol-Pol et al., 2018; Sunderhaus and Kretzschmar, 2016; Wittmann et al., 2001). To investigate how protein reabsorption in nephrocytes affects *Drosophila* locomotor capacities and aging, we knocked down the *dCubilin* gene or the *dAMN* gene specifically in nephrocytes and examined their effect on brain aging. As shown in Fig. 5A,B, compared to the control group, *dAMN* or *dCubilin* knockdown in nephrocytes resulted in increased vacuole formation in brains. The number of vacuoles per brain in each group was calculated. Compared to control flies, 30-day-old flies with *dCubilin* or *dAMN* knockdown showed significantly increased vacuole formation in brain (9.3 for *dAMN*-RNAi, 11.4 for *dCubilin*-RNAi and 6.4 for control flies; Fig. 5B). Brains of 15-day-old *dCubilin* knockdown flies also showed a significantly increased number of vacuoles compared to control flies (6.5 for *dCubilin*-RNAi and 3.5 for control flies; Fig. 5B). We also found that the size of vacuoles in the brains significantly increased with aging (Fig. 5C). The size of vacuoles (>20 µm) in the brains of 15-day-old *dCubilin* or dAMN knockdown flies dramatically increased compared to control flies (70.5% for *dCubilin*-RNAi, 69.4% for *dAMN*-RNAi and 55.5% for control flies). Brains of 30-day-old *dCubilin* or *dAMN* knockdown flies also exhibited significantly larger vacuoles (>50 µm) compared to control flies (47.1% for *dCubilin*-RNAi, 48.9% for *dAMN*-RNAi and 33.8% for control flies). On the contrary, overexpression of dAMN in nephrocytes led to decreased vacuole formation in the brains of aging flies compared to their respective control groups (4.5 for dAMN-overexpression flies, *P*<0.01) (Fig. 5A,B). Compared to the control group, the size of vacuoles (>50 µm) in the brains of dAMN overexpression flies also dramatically decreased (18.2% for dAMN-OE and 30.5% for control flies in the 15-day-old flies, and 22.9% for dAMN-OE and 33.8% for control flies in the 30-day-old flies) (Fig. 5C).

**Fig. 6.**
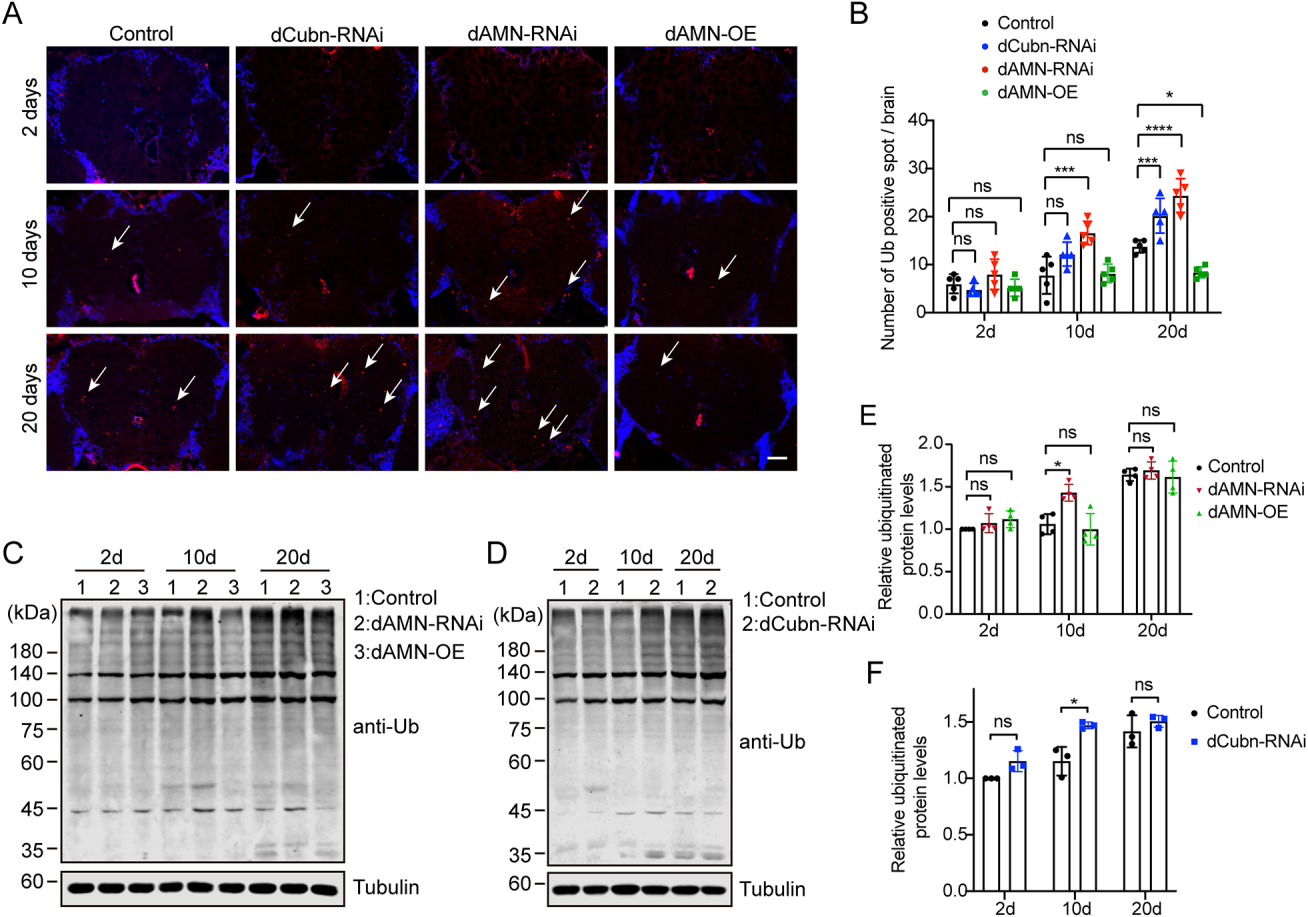
**dCubilin- or dAMN-mediated protein reabsorption in nephrocytes regulated the aggregation of ubiquitinated proteins in *Drosophila* brain.** (A,B) Representative micrographs and quantification of immunofluorescence staining for ubiquitinated proteins in brains (arrows indicate ubiquitinated proteins). Scale bar: 50 μm. *n*=5. (C,D) Representative western blot images show the levels of ubiquitinated proteins in brains in different groups, as indicated. (E,F) Quantitative data of western blot. Data were acquired from at least three individual replicates. Data in E were representative of four independent experiments, and data in F were representative of three independent experiments. Data are mean±s.d. *P<0.05; ***P<0.001; ****P<0.0001; ns, not significant [one-way ANOVA (B,E); unpaired and two-tailed Student's *t*-test (F)].

Accumulation of intracellular damaged proteins is a hallmark of aging. Ubiquitinated proteins are accumulated in aging tissues because of decreased proteasome and/or autophagy activity. To investigate how protein reabsorption in nephrocytes affects *Drosophila* brain aging, we examined the ubiquitinated protein level in the brain using both immunofluorescence staining and western blot analysis. As shown in Fig. 6A,B, compared to the control group, the ubiquitinated protein spots dramatically increased in brains of aging flies with nephrocyte-specific RNAi knockdown of *dAMN* or *dCubilin* (16.6 for *dAMN*-RNAi, 12.2 for *dCubilin*-RNAi and 7.8 for control in the 10-day-old flies; 24.4 for *dAMN*-RNAi, 20.2 for *dCubilin*-RNAi and 13.8 for control in the 20-day-old flies). However, the amount of ubiquitinated proteins significantly decreased in brains of aging flies overexpressing dAMN (8.4 ubiquitinated protein spots per brain in the 20-day-old flies) compared to control flies (Fig. 6A,B). We also examined the ubiquitinated protein level in the whole-head lysates using western blot analysis with anti-poly-ubiquitin antibody. As shown in Fig. 6C-F, compared to the control group, ubiquitinated proteins significantly increased in the whole-head lysates of 10-day-old flies with nephrocyte-specific RNAi knockdown of *dAMN* or *dCubilin*. However, western blot did not detect any difference in the whole-head lysates of 20-day-old flies with nephrocyte-specific RNAi knockdown of *dAMN* or *dCubilin* compared to the control flies (Fig. 6C-F). Additionally, we did not detect any difference in the whole-head lysates of dAMN overexpression flies either (Fig. 6C,E), perhaps because of the interference of the ubiquitinated proteins in other parts of the head, such as the eyes and cuticles.

### dCubilin- or dAMN-mediated protein reabsorption in nephrocytes affects *Drosophila* muscle proteostasis and aging

**Fig. 7.**
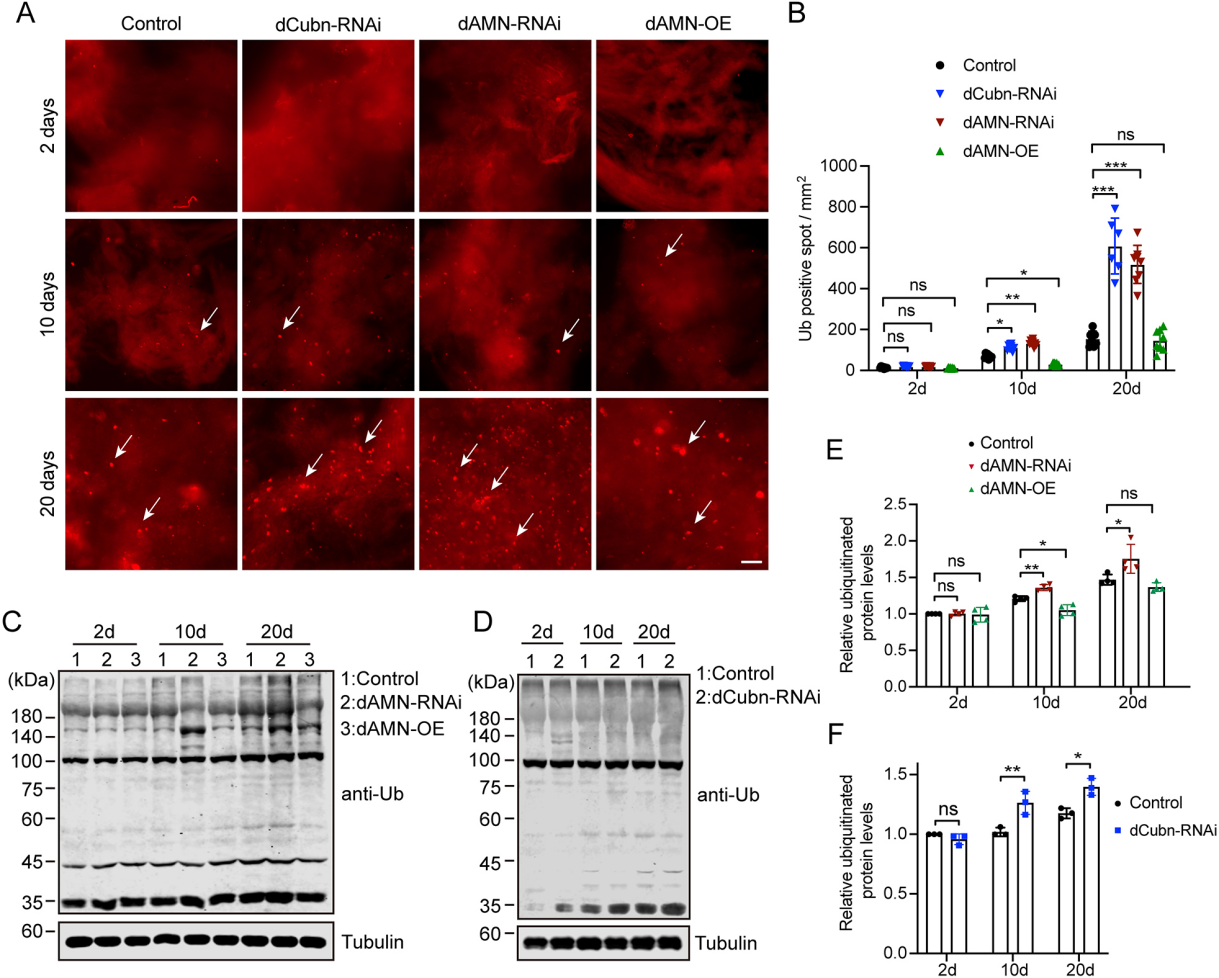
**dCubilin- or dAMN-mediated protein reabsorption in nephrocytes impacts the accumulation of ubiquitinated proteins in *Drosophila* muscle.** (A,B) Representative micrographs and quantification of immunofluorescence staining for ubiquitinated proteins in muscles (arrows indicate ubiquitinated proteins). Scale bar: 50 μm. *n*=6. (C,D) Representative western blot images show the levels of ubiquitinated proteins in muscles in different groups, as indicated. (E,F) Quantitative data of western blot. Data were acquired from at least three individual replicates. Data in E were representative of four independent experiments, and data in F were representative of three independent experiments. Data are mean±s.d. *P<0.05; **P<0.01; ***P<0.001; ns, not significant [one-way ANOVA (B,E); unpaired and two-tailed Student's *t*-test (F)].

Previous studies suggested that inter-organ proteostasis networks (tele-proteostasis) coordinate the response to proteotoxic insult at the tissue/organ level (Fernando et al., 2019; Kaushik and Cuervo, 2015; Korovila et al., 2017; Morimoto, 2020; Morimoto and Cuervo, 2014). dCubilin- and dAMN-mediated protein reabsorption in nephrocytes affects proteostasis in *Drosophila* hemolymph. As protein reabsorption in nephrocytes affects *Drosophila* locomotor capacities and aging, we speculated that protein accumulation in hemolymph might disturb proteostasis in muscles, thus affecting locomotor activity. To test this hypothesis, we measured the ubiquitinated protein level in muscle tissue using immunofluorescence staining. As shown in Fig. 7, compared to the control group, ubiquitinated proteins significantly accumulated in muscles of 10-day- or 20-day-old flies with nephrocyte-specific *dAMN* or *dCubilin* RNAi knockdown (Fig. 7A,B). On the contrary, ubiquitinated protein levels were decreased in muscles of 10-day-old flies with nephrocyte-specific dAMN overexpression (Fig. 7A,B). Compared to control flies, the amount of ubiquitinated proteins was significantly increased in 10-day-old flies with *dCubilin* or *dAMN* knockdown (133.29 spots/mm^2^ for *dAMN*-RNAi, 104.55 spots/mm^2^ for *dCubilin*-RNAi and 38.61 spots/mm^2^ for control flies; *P*<0.05; Fig. 7B). Muscles of 20-day-old *dCubilin*- or *dAMN*-deficient flies showed a significantly greater number of ubiquitinated proteins than control flies (608.48 spots/mm^2^ for *dCubilin*-RNAi, 518.70 spots/mm^2^ for *dAMN*-RNAi and 156.14 spots/mm^2^ for control flies; Fig. 7B). We further examined the ubiquitinated protein level in the muscles of flies overexpressing dAMN proteins specifically in nephrocytes. As shown in Fig. 7A,B, compared to the control group, dAMN overexpression in nephrocytes resulted in decreased levels of ubiquitinated proteins in muscle tissue of 10-day-old flies (21.47 spots/mm^2^ for dAMN-OE and 38.61 spots/mm^2^ for control flies; Fig. 7B). Muscle tissue of 20-day-old flies overexpressing dAMN in nephrocytes did not show a decreased number of ubiquitinated proteins compared to control flies (146.93/mm^2^ for dAMN-OE flies and 156.14/mm^2^ for control flies; Fig. 7B).

We also examined the ubiquitinated protein level in the dissected muscle lysates using western blot analysis with anti-poly-ubiquitin antibody. As shown in Fig. 7C-F, compared to the control group, ubiquitinated proteins significantly increased in the dissected muscle lysates of 10-day- or 20-day-old flies with nephrocyte-specific RNAi knockdown of *dAMN* or *dCubilin*. As shown in Fig. 7C,E, total ubiquitinated proteins significantly increased in the dissected muscle lysates of 10-day-old flies with nephrocyte-specific overexpression of dAMN compared to the control flies of the same age. Taken together, these results strongly suggest that enhanced protein reabsorption in nephrocytes improves proteostasis in muscle tissues and delays *Drosophila* muscle aging.

### dCubilin- or dAMN-mediated protein reabsorption in nephrocytes affects *Drosophila* proteasome activity

**Fig. 8.**
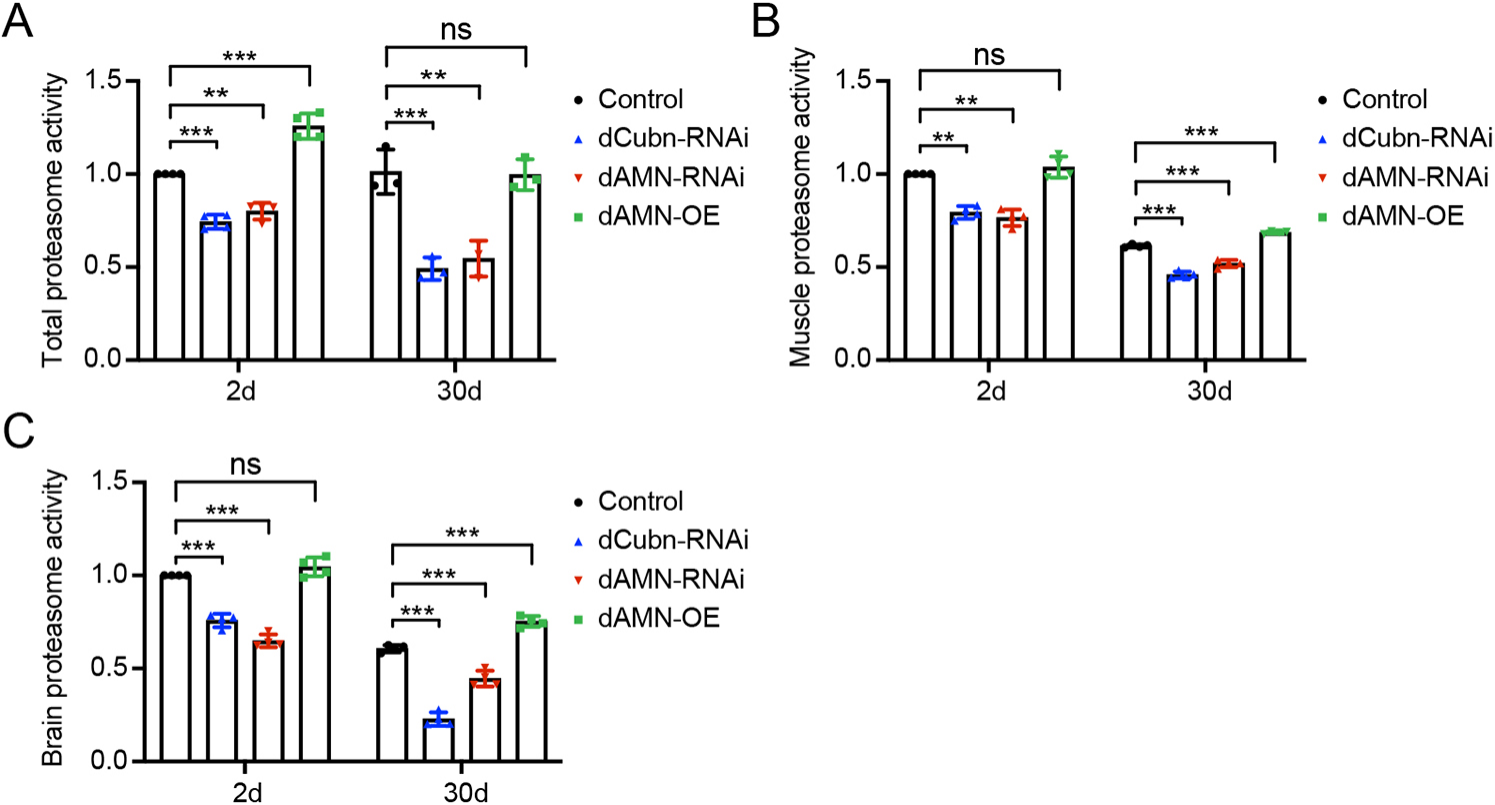
**dCubilin- or dAMN-mediated protein reabsorption in nephrocytes affects proteasome activity.** Quantitative data of proteasome activity of total protein extract from 2-day- and 30-day-old flies with different genotypes, as indicated. (B) Quantification of proteasome activity in muscle tissue of 2-day- and 30-day-old flies with different genotypes, as indicated. (C) Quantitative data of proteasome activity in brain tissue of 2-day- and 30-day-old flies with different genotypes, as indicated. Data are mean±s.d. and representative of at least three independent experiments. **P<0.01; ***P<0.001; ns, not significant (one-way ANOVA).

dCubilin and dAMN-mediated protein reabsorption in nephrocytes affects proteostasis in *Drosophila* hemolymph, muscles and brain. To investigate the molecular mechanism through which dCubilin and dAMN-mediated protein reabsorption in nephrocytes affects proteostasis in muscles and brain, we measured the proteasome activity in nephrocyte-specific *dAMN* or *dCubilin* knockdown and overexpressing flies. As shown in Fig. 8A, compared to the control group, proteasome activity was significantly decreased in total fly extract of 2-day- and 30-day-old flies with *dAMN* or *dCubilin* RNAi knockdown in nephrocytes. dAMN overexpression in nephrocytes increased proteasome activity in total fly extract of 2-day-old flies, but not in 30-day-old flies (Fig. 8A). To test whether proteostasis in hemolymph affects proteasome activity in muscle tissue, we also measured proteasome activity changes in muscle tissues. Compared to the control group, proteasome activity in muscle tissue was significantly decreased in 2-day- and 30-day-old flies with nephrocyte-specific RNAi knockdown of *dAMN* or *dCubilin*. On the contrary, proteasome activity was increased in muscle tissues of 30-day-old flies with nephrocyte-specific dAMN overexpression (Fig. 8B). We further measured the changes of proteasome activity in brain tissues. Proteasome activity in the head was significantly decreased in 2-day- and 30-day-old flies with nephrocyte-specific RNAi knockdown of *dAMN* or *dCubilin* compared to the control group, but it was increased in the head of 30-day-old flies with nephrocyte-specific dAMN overexpression (Fig. 8C).

## DISCUSSION

The proteostasis network coordinates protein homeostasis intracellularly, between cells and across organs, and might lead to common age-associated diseases when it is disrupted (Fernando et al., 2019; Kaushik and Cuervo, 2015; Morimoto, 2020; Santra et al., 2019). Tele-proteostasis has been proposed as a mechanism for crosstalk between the proteostasis networks in different organs as a means to coordinate their response (Kaushik and Cuervo, 2015). However, more evidence is needed to establish the existence of the tele-proteostasis network at the whole-organism level. The *Drosophila* nephrocyte shares remarkable similarities with the renal proximal tubule in mammals and performs similar protein reabsorption functions (Na and Cagan, 2013; Weavers et al., 2009; Zhang et al., 2013a). *Drosophila* homologs of mammalian Cubilin and Amnionless (dCubilin and dAMN, respectively) have been shown to be required for nephrocyte protein reabsorption *in vivo* (Zhang et al., 2013a). In this study, using *Drosophila* as a model system, we uncovered that dCubilin- and dAMN-mediated protein reabsorption in *Drosophila* nephrocytes modulates longevity by regulating proteostasis in muscle and brain tissues via a tele-proteostasis mechanism.

### dCubilin- or dAMN-mediated protein reabsorption in nephrocytes modulates *Drosophila* lifespan and regulates hemolymph proteostasis

Our previous study showed that dCubilin- and dAMN-mediated protein reabsorption in *Drosophila* nephrocytes is essential for toxin removal from hemolymph (Zhang et al., 2013a). However, gaps in our knowledge regarding the exact contribution of nephrocytes and a potential role in tele-proteostasis remain. Our study showed that enhanced protein reabsorption in nephrocyte-specific dAMN-overexpressing flies dramatically extends lifespan, whereas decreased protein reabsorption due to nephrocyte-specific *dCubilin* or *dAMN* RNAi knockdown shortens lifespan in flies. We also observed that the effect of nephrocyte-specific dAMN overexpression or *dAMN* or *dCubilin* RNAi knockdown on lifespan in female flies was greater than in male flies. However, we did not find a significant difference between male and female flies with regards to brain or muscle aging in dAMN overexpression or *dAMN* or *dCubilin* RNAi knockdown flies. Aging is a complicated process regulated by multiple factors. Proteostasis is one of these factors, but other factors, such as hormones, may contribute to the gender difference of the effect of protein reabsorption on lifespan.

In aged wild-type flies, proteins accumulated in hemolymph under normal conditions. Nephrocyte-specific *dCubilin* or *dAMN* RNAi knockdown led to increased hemolymph protein accumulation, whereas nephrocyte-specific overexpression of dAMN resulted in decreased hemolymph protein accumulation. In the future, it will be interesting to determine the composition of the hemolymph using mass spectrometry to identify the major components that show drastic changes in nephrocyte-specific *dAMN* knockdown or overexpressing flies across the lifespan, which can then be compared to the human findings to confirm the foundation and boundaries of *Drosophila* as an *in vivo* model system to study tele-proteostasis in general and in the context of aging.

### dCubilin- or dAMN-mediated protein reabsorption in nephrocytes impacts aging and proteostasis in *Drosophila* brain and muscle tissues

Reduced insulin/insulin-like growth factor signaling extends lifespan in *Drosophila* (Augustin et al., 2018; Bai et al., 2012; Kannan and Fridell, 2013; Partridge, 2001). *Drosophila* insulin-like peptide-6 (dilp6) expression from fat body represses the secretion of *Drosophila* insulin-like peptide-2 (dilp2) from the brain into hemolymph and extends lifespan, suggesting that hemolymph composition is critical for lifespan regulation (Bai et al., 2012). Previous studies have suggested that inter-organ proteostasis networks (tele-proteostasis) coordinate the response of tissues or organs to proteotoxic insults (Fernando et al., 2019; Kaushik and Cuervo, 2015; Korovila et al., 2017; Morimoto, 2020). The formation of vacuoles in the *Drosophila* brain is a hallmark of brain aging and neurodegenerative diseases (Deng et al., 2020; Ordonez et al., 2018; Wittmann et al., 2001). Our results showed that RNAi knockdown of *dAMN* or *dCubilin* in nephrocytes led to increased vacuole formation in brains, and overexpression of dAMN in nephrocytes resulted in decreased vacuole formation in the brains of 30-day-old flies, indicating that dCubilin- or dAMN-mediated protein reabsorption in nephrocytes could modulate brain aging via tele-proteostasis.

Ubiquitinated proteins are accumulated in aging tissues as a result of decreased proteasome and/or autophagy activities (Nguyen et al., 2019; Tsakiri et al., 2013). Our study showed that ubiquitinated proteins significantly accumulate in the brains of 10-day- and 20-day-old flies with *dAMN* or *dCubilin* RNAi knockdown in nephrocytes. In contrast, overexpression of dAMN in nephrocytes led to decreased accumulation of ubiquitinated proteins in the brains of 10-day-old flies. Our results also showed that ubiquitinated proteins significantly accumulated in the muscles of 10-day- and 20-day-old flies with nephrocyte-specific *dAMN* or *dCubilin* RNAi knockdown, whereas ubiquitinated protein level decreased in the muscles of 10-day-old flies with nephrocyte-specific overexpression of dAMN. Enhancing protein reabsorption in nephrocytes improves proteostasis in brain and muscle tissue, and delays *Drosophila* muscle aging. Protein reabsorption defects in nephrocytes leads to ubiquitinated protein accumulation in brain and muscle tissue, and accelerates *Drosophila* brain and muscle aging. These results suggest that dCubilin- or dAMN-mediated protein reabsorption in nephrocytes regulates proteostasis in brain and muscle tissues via the tele-proteostasis mechanism.

### dCubilin- or dAMN-mediated protein reabsorption in nephrocytes affects *Drosophila* proteasome activity in the brain and muscle tissues

The ubiquitin-proteasome system is responsible for the removal of both normal and damaged proteins in different cell types. It has been shown that proteasome activity decreases during aging and senescence (Ben-Zvi et al., 2009; Demontis and Perrimon, 2010). Enhancement of proteasome levels and activity in *Drosophila* results in lifespan extension and increased resistance to oxidative stress (Nguyen et al., 2019). In line with those findings, our study showed that dCubilin- or dAMN-mediated protein reabsorption in nephrocytes affects proteasome activity in the whole body, brain and muscle tissues. The proteasome activity was significantly decreased in the head and muscle tissue of 2-day- and 30-day-old flies with nephrocyte-specific *dAMN* or *dCubilin* RNAi knockdown, whereas proteasome activity increased in the head and muscle tissue of 30-day-old flies with nephrocyte-specific dAMN overexpression but not in 2-day-old flies. The changes in proteasome activity are correlated with the alteration of proteostasis in the brain and muscle tissues. Together, these results suggest that the impact of altered proteostasis in hemolymph on proteasome activity in brain and muscle tissue is a long-term effect, thereby supporting a coordinating role for the tele-proteostasis network.

Our results showed that dCubilin- or dAMN-mediated protein reabsorption in nephrocytes impacts proteostasis in hemolymph. Protein accumulation increases in *Drosophila* hemolymph with aging, indicating that dCubilin- or dAMN-mediated protein reabsorption in nephrocytes decreases with aging. Our previous study showed that dCubilin and dAMN are essential for the maintenance of nephrocyte ultrastructures (Zhang et al., 2013a). *Drosophila* pericardial nephrocytes also undergo dramatic histological degeneration upon aging, such as the loss of slit diaphragms, the degeneration of labyrinth channels and the enrichment of lysosomes with electron-dense materials (Psathaki et al., 2018). All these structural alterations could also lead to decreased protein reabsorption in nephrocytes. It could be very useful to distinguish these different possibilities if in the future, when antibodies become available, we can clearly demonstrate whether the expression levels of dCubilin or dAMN decreases with aging. Insect nephrocyte functions are equivalent to the vertebrate glomerulus and the renal proximal tubule for both filtration and protein reabsorption, and the Malpighian tubules are similar to the loop of Henle and the renal distal tubule, which are mainly used for water and salt reabsorption (Cagan, 2011). Our results showed that dCubilin- or dAMN-mediated protein reabsorption in nephrocytes modulates *Drosophila* lifespan by impacting proteostasis in hemolymph, brain and muscle tissues. However, we cannot exclude the possibility that dCubilin- or dAMN-mediated protein reabsorption in nephrocytes could indirectly impact water homeostasis because of water retention caused by protein accumulation, which further affects the aging process.

There are some limitations in our current study. As we all know, subtle differences in genetics can cause lifespan differences. In our current study, we used the UAS/Gal4 system to knockdown *dAMN* and *dCubilin* or overexpress dAMN in nephrocytes, and examined the effect of protein reabsorption on lifespan. There could be some potential genetic background differences between different genotypes, which could affect *Drosophila* lifespan. The GeneSwitch system is a modified Gal4/UAS system, whereby transgene expression is induced in *Drosophila* by adding RU486 to food. The GeneSwitch system is widely used in *Drosophila* aging and behavioral studies to avoid confounding effects related to genetic background mutations. In this study, we did not use the GeneSwitch system because no nephrocyte-specific GS-Gal4 is currently available. In the future, it will be better for us to generate a new nephrocyte-specific GS-Gal4 line and compare the phenotype difference between *dAMN* knockdown flies using Dot-Gal4 and GS-Gal4.

In summary, in this study, we showed that dCubilin- or dAMN-mediated protein reabsorption from hemolymph by *Drosophila* nephrocytes modulates longevity by regulating proteostasis in muscle and brain tissues via a tele-proteostasis network. Our study provides solid evidence for the existence of a tele-proteostasis network that coordinates proteostasis across different organs in *Drosophila*.

## MATERIALS AND METHODS

### Fly strains

Flies were reared on a diet containing 1% agar, 6.25% cornmeal, 6.25% molasses and 3.5% Red Star yeast at 25°C. Flies were kept in 12-h light-dark cycles (with an average light intensity of ∼1500 lx). All UAS-Gal4 crosses were performed at 25°C and virgins were always collected from the pMHC-ANF-RFP,Hand-GFP; Dot-Gal4 driver line. Dot-Gal4 was obtained from the Bloomington *Drosophila* Stock Center (BDSC). The following flies were used: UAS-dCubilin^RNAi^ (P{TRiP.JF03118}attP2, BDSC); UAS-dCubilin^RNAi^ [P{GD6458}v14613, Vienna *Drosophila* Resource Center (VDRC)]; UAS-dAMN^RNAi^ (P{KK103998} v104099, VDRC); and UAS-dAMN^RNAi^ (P{GD781}v2495, VDRC). Generation of MHC-ANF-RFP (pMAR), UAS-dAMN and UAS-hAMN have been described previously (Zhang et al., 2013a,b). Hand-GFP was used to label nephrocytes at all developmental stages.

### RNAi-based nephrocyte functional analysis

Details of the functional analysis procedures have been described previously (Zhang et al., 2013b). Briefly, ten virgins of pMHC-ANF-RFP,Hand-GFP; Dot-Gal4 flies were crossed with five males of UAS-RNAi transgenic lines in vials at 25°C. The progenies of PH,pMAR; Dot-Gal4 virgins crossed with W^1118^ male flies were used as controls in all the following experiments. Two days after crossing, flies were transferred to small collection cages with grape juice agar plates for 24 h at 25°C. Collected embryos were aged for 48 h at 29°C, and then second and third instar larvae were subjected to examination of the RFP signal in pericardial nephrocytes using a Leica TCS SP8 confocal microscope with a 20× objective. The fluorescence intensity of secreted atrium natriuretic factor (ANF)-RFP that accumulated in pericardial nephrocytes was quantified in three nephrocytes per fly. The pericardial nephrocytes were selected randomly.

### Immunofluorescence staining and confocal imaging analysis

Indirect muscles or heads were dissected and fixed in freshly prepared 4% paraformaldehyde in PBS with 0.1% Triton X-100 (PBS-T 0.1%) for 30 min, rinsed with PBS with 0.5% Triton X-100 (PBS-T 0.5%) and blocked for 1 h at room temperature with 5% bovine serum albumin. The samples were then incubated for 24 h in 1:200 primary anti-ubiquitin rabbit pAb (PTM-Bio; PTM-1106) antibody overnight at 4°C, rinsed five times in PBS-T 0.5% and incubated for 2 h in Cy3-AffiniPure donkey anti-rabbit IgG (Jackson ImmunoResearch, Life Technologies, 711-165-152, 1:200). Samples were then rinsed six times with PBS-T 0.5% and mounted on microscope slides in Vectashield mounting medium with DAPI (Vector Laboratories, Burlingame, California). Images were taken using an Olympus BX61 microscope with all parameters held constant throughout. Ubiquitinated protein level was evaluated by counting the fluorescence spots in a specifically designated area.

### Hemolymph protein extraction and SDS-PAGE analysis

Twenty male and 20 female adult flies of a certain age were anesthetized with carbon dioxide and a hole was gently punched using forceps. The flies were placed into a 100 µm strainer that was placed on top of a 1.5 ml tube containing 10 µl 2× SDS loading buffer. The samples were then centrifuged at 8600 ***g*** for 5 min and the supernatant was collected. Total protein extracts were subjected to SDS-PAGE analysis and quantified using ImageJ.

### Fruit fly longevity assay

Virgins of pMHC-ANF-RFP, Hand-GFP; Dot-Gal4 flies were crossed with males of UAS-RNAi transgenic lines in bottles at 25°C. The F1 progenies were used for lifespan analysis. The progenies of pMHC-ANF-RFP, Hand-GFP; Dot-Gal4 virgins crossed with W^1118^ male flies were used as controls. Lifespan was measured using at least ten vials of ten mated males or females. Males or females of a given genotype were housed in 8-oz round-bottom polypropylene vials containing 1 ml of food. Flies were tapped to the bottom of the vials without anesthesia for food exchange and mortality was recorded every 2-3 days. Lifespan graphs were plotted using survival curves. Some flies that escaped when we transferred flies from one vial to another vial were censored in our analyses.

### Negative geotaxis assay

A negative geotaxis assay was used to measure the locomotor activity of flies following an established protocol (Cao et al., 2017; Madabattula et al., 2015). For each genotype tested, ten groups of ten flies were transferred without anesthesia into empty vials with a mark 8 cm from the bottom and rested on the table for 1 min. Each vial was tapped three times in rapid succession to initiate a negative geotaxis response. Climbing activity of the flies in the vials was recorded and the CI was calculated by dividing the number of flies passing the 8-cm line mark by total fly numbers. CI_50_ represents the age of flies with 50% of flies passing the 8-cm line mark.

### Hematoxylin and Eosin staining, and brain vacuole analysis

Flies of each genotype were allowed to reach a specified age and heads were processed as described previously (Reenan and Rogina, 2008). Briefly, the fly heads were dissected at indicated time points, immediately fixed in 4% neutral buffered formalin at 4°C for 12 h and dehydrated using graded ethanol, and then paraffin-embedded and sectioned (3 µm). After slides were deparaffinized and rehydrated, sections were stained using a Hematoxylin and Eosin staining kit (Solarbio) according to the manufacturer's instructions. Microscopic images were taken at the same level of the brain using an Olympus BX61 upright microscope. The size of vacuoles was measured using ImageJ and divided into four different sizes according to the area of vacuoles (5-20 μm^2^, 20-50 μm^2^, 50-150 μm^2^ and more than 150 μm^2^). The percentage of different size vacuoles in each fly was calculated.

### Western blot analysis

Ubiquitinated protein expression was analyzed by western blotting. Briefly, dissected muscle or head tissue was washed twice with PBS and was then lysed and homogenized in lysis buffer, and their protein concentrations were measured using a bicinchoninic acid assay. The homogenates were then subjected to SDS-PAGE electrophoresis. After electrophoresis, the proteins were transferred to a nitrocellulose filter membrane (Merck Millipore), blocked in blocking buffer (5% skimmed milk) for 1 h at room temperature and then incubated with primary antibodies overnight at 4°C, and a secondary antibody for 1 h at room temperature. The antigen-antibody complexes were visualized automatically using an Odyssey CLx Near-infrared imaging system (LI-COR, USA). The antibodies used were as follows: anti-α-tubulin (1:2000; Beyotime, AT819); anti-ubiquitin rabbit pAb (1:5000; PTM-Bio, PTM-1106); goat anti-mouse IRDye 800CW (1:100,000; LI-COR, 926-32210); and goat anti-rabbit IRDye 800CW (1:100,000; LI-COR, 926-32211).

### Proteasome activity assay

20S proteasome activity was measured using AMC-Suc-LLVY substrate (LifeSensors, PS500). HEPES (50 mM, pH 7.8), 10 mM NaCl, 1.5 mM MgCl_2_, 1 mM EDTA and 250 mM sucrose were used to prepare basic buffer. The whole fly, dissected muscle or head tissue were washed twice with PBS and then lysed and homogenized in lysis buffer [(basic buffer containing 1 mM dithiothreitol (DTT)]. Cell debris was removed by centrifugation at 10,000 ***g*** for 10 min and the supernatants were used for the proteasome activity assay. Protein concentration was measured by Bradford assay and adjusted to 3 μg/μl. To measure proteasome activity, 5 µl lysis buffer or protein lysate was added to a 96-well plate, along with 200 µl of assay buffer (basic buffer containing 5 mM DTT, 2 mM ATP and 100 µM AMC-Suc-LLVY substrate) as the blank or sample pore. The reaction mixture was incubated for 1 h at 37°C. Proteasome activity was measured by monitoring the intensity of fluorescence (excitation, 380 nm; emission, 460 nm) using a PerkinElmer EnSpire Multilabel Reader 2300 (PerkinElmer, Waltham, MA, USA). Proteasome activity in flies with different genotypes and ages were compared to that of 2-day-old wild-type flies.

### Statistical analyses

Statistical analysis of the data was performed using SPSS version 24.0 (SPSS Inc, Chicago, IL, USA) and GraphPad Prism version 8.0 software (GraphPad Software, La Jolla, CA, USA). To determine statistical differences between multiple genotypes, a one-way ANOVA test was applied, followed by a Bonferroni test. When the variances were not equal, *P* values were calculated using one-way ANOVA analysis and Dunnett's T3. When only two groups were compared and data did not follow a normal distribution as assessed by a d'Agostino–Pearson omnibus test, statistical significance was determined using a Mann–Whitney U non-parametric test (e.g. in the quantification of the protein accumulation in the hemolymph). Lifespan graphs were plotted using survival curves. For statistical analysis, a log-rank test (Mantel–Cox) was applied to determine significant differences between survival curves. Some flies that escaped when we transferred flies from one vial to another vial were censored in our analyses. *P*<0.05 was considered significant.

## References

[DMM047464C1] Augustin, H., McGourty, K., Allen, M. J., Adcott, J., Wong, C. T., Boucrot, E. and Partridge, L. (2018). Impact of insulin signaling and proteasomal activity on physiological output of a neuronal circuit in aging *Drosophila* melanogaster. *Neurobiol. Aging* 66, 149-157. 10.1016/j.neurobiolaging.2018.02.027 29579685 PMC5933513

[DMM047464C2] Bai, H., Kang, P. and Tatar, M. (2012). *Drosophila* insulin-like peptide-6 (dilp6) expression from fat body extends lifespan and represses secretion of *Drosophila* insulin-like peptide-2 from the brain. *Aging Cell* 11, 978-985. 10.1111/acel.12000 22935001 PMC3500397

[DMM047464C3] Ben-Zvi, A., Miller, E. A. and Morimoto, R. I. (2009). Collapse of proteostasis represents an early molecular event in Caenorhabditis elegans aging. *Proc. Natl. Acad. Sci. USA* 106, 14914-14919. 10.1073/pnas.0902882106 19706382 PMC2736453

[DMM047464C4] Cabirol-Pol, M.-J., Khalil, B., Rival, T., Faivre-Sarrailh, C. and Besson, M. T. (2018). Glial lipid droplets and neurodegeneration in a *Drosophila* model of complex I deficiency. *Glia* 66, 874-888. 10.1002/glia.23290 29285794

[DMM047464C5] Cagan, R. L. (2011). The *Drosophila* nephrocyte. *Curr. Opin. Nephrol. Hypertens.* 20, 409-415. 10.1097/MNH.0b013e328347ae02 21610496

[DMM047464C6] Cao, W., Song, L., Cheng, J., Yi, N., Cai, L., Huang, F.-D. and Ho, M. (2017). An automated rapid iterative negative geotaxis assay for analyzing adult climbing behavior in a *Drosophila* model of neurodegeneration. *J. Vis. Exp.* 56507. 10.3791/56507 PMC5752225 28931001

[DMM047464C7] Cheon, S. Y., Kim, H., Rubinsztein, D. C. and Lee, J. E. (2019). Autophagy, cellular aging and age-related human diseases. *Exp. Neurobiol.* 28, 643-657. 10.5607/en.2019.28.6.643 31902153 PMC6946111

[DMM047464C8] Chondrogianni, N., Georgila, K., Kourtis, N., Tavernarakis, N. and Gonos, E. S. (2015). 20S proteasome activation promotes life span extension and resistance to proteotoxicity in Caenorhabditis elegans. *FASEB J.* 29, 611-622. 10.1096/fj.14-252189 25395451 PMC4314225

[DMM047464C9] Conboy, M. J., Conboy, I. M. and Rando, T. A. (2013). Heterochronic parabiosis: historical perspective and methodological considerations for studies of aging and longevity. *Aging Cell* 12, 525-530. 10.1111/acel.12065 23489470 PMC4072458

[DMM047464C10] Conese, M., Carbone, A., Beccia, E. and Angiolillo, A. (2017). The fountain of youth: a tale of parabiosis, stem cells, and rejuvenation. *Open Med.* 12, 376-383. 10.1515/med-2017-0053 PMC5662775 29104943

[DMM047464C11] Cuervo, A. M. and Wong, E. (2014). Chaperone-mediated autophagy: roles in disease and aging. *Cell Res.* 24, 92-104. 10.1038/cr.2013.153 24281265 PMC3879702

[DMM047464C12] Demontis, F. and Perrimon, N. (2010). FOXO/4E-BP signaling in *Drosophila* muscles regulates organism-wide proteostasis during aging. *Cell* 143, 813-825. 10.1016/j.cell.2010.10.007 21111239 PMC3066043

[DMM047464C13] Deng, P., Khan, A., Jacobson, D., Sambrani, N., McGurk, L., Li, X., Jayasree, A., Hejatko, J., Shohat-Ophir, G., O'Connell, M. A. et al. (2020). Adar RNA editing-dependent and -independent effects are required for brain and innate immune functions in *Drosophila*. *Nat. Commun.* 11, 1580. 10.1038/s41467-020-15435-1 32221286 PMC7101428

[DMM047464C14] Eggel, A. and Wyss-Coray, T. (2014). A revival of parabiosis in biomedical research. *Swiss Med. Wkly.* 144, w13914. 10.4414/smw.2014.13914 24496774 PMC4082987

[DMM047464C15] Fernando, R., Drescher, C., Nowotny, K., Grune, T. and Castro, J. P. (2019). Impaired proteostasis during skeletal muscle aging. *Free Radic. Biol. Med.* 132, 58-66. 10.1016/j.freeradbiomed.2018.08.037 30194981

[DMM047464C16] Guillou, A., Troha, K., Wang, H., Franc, N. C. and Buchon, N. (2016). The *Drosophila* CD36 homologue croquemort is required to maintain immune and gut homeostasis during development and aging. *PLoS Pathog.* 12, e1005961. 10.1371/journal.ppat.1005961 27780230 PMC5079587

[DMM047464C17] Kannan, K. and Fridell, Y.-W. C. (2013). Functional implications of *Drosophila* insulin-like peptides in metabolism, aging, and dietary restriction. *Front. Physiol.* 4, 288. 10.3389/fphys.2013.00288 24137131 PMC3797364

[DMM047464C18] Kaushik, S. and Cuervo, A. M. (2015). Proteostasis and aging. *Nat. Med.* 21, 1406-1415. 10.1038/nm.4001 26646497

[DMM047464C19] Klaips, C. L., Jayaraj, G. G. and Hartl, F. U. (2018). Pathways of cellular proteostasis in aging and disease. *J. Cell Biol.* 217, 51-63. 10.1083/jcb.201709072 29127110 PMC5748993

[DMM047464C20] Korovila, I., Hugo, M., Castro, J. P., Weber, D., Höhn, A., Grune, T. and Jung, T. (2017). Proteostasis, oxidative stress and aging. *Redox Biol.* 13, 550-567. 10.1016/j.redox.2017.07.008 28763764 PMC5536880

[DMM047464C21] Labbadia, J. and Morimoto, R. I. (2015). The biology of proteostasis in aging and disease. *Annu. Rev. Biochem.* 84, 435-464. 10.1146/annurev-biochem-060614-033955 25784053 PMC4539002

[DMM047464C22] Lehallier, B., Gate, D., Schaum, N., Nanasi, T., Lee, S. E., Yousef, H., Moran Losada, P., Berdnik, D., Keller, A., Verghese, J. et al. (2019). Undulating changes in human plasma proteome profiles across the lifespan. *Nat. Med.* 25, 1843-1850. 10.1038/s41591-019-0673-2 31806903 PMC7062043

[DMM047464C23] Madabattula, S. T., Strautman, J. C., Bysice, A. M., O'Sullivan, J. A., Androschuk, A., Rosenfelt, C., Doucet, K., Rouleau, G. and Bolduc, F. (2015). Quantitative analysis of climbing defects in a *Drosophila* model of neurodegenerative disorders. *J. Vis. Exp.* e52741. 10.3791/52741 26132637 PMC4544889

[DMM047464C24] Madeo, F., Zimmermann, A., Maiuri, M. C. and Kroemer, G. (2015). Essential role for autophagy in life span extension. *J. Clin. Invest.* 125, 85-93. 10.1172/JCI73946 25654554 PMC4382258

[DMM047464C25] Morimoto, R. I. (2020). Cell-nonautonomous regulation of proteostasis in aging and disease. *Cold Spring Harb. Perspect. Biol.* 12, a034074. 10.1101/cshperspect.a034074 30962274 PMC7111247

[DMM047464C26] Morimoto, R. I. and Cuervo, A. M. (2014). Proteostasis and the aging proteome in health and disease. *J. Gerontol. A Biol. Sci. Med. Sci.* 69 Suppl. 1, S33-S38. 10.1093/gerona/glu049 24833584 PMC4022129

[DMM047464C27] Na, J. and Cagan, R. (2013). The *Drosophila* nephrocyte: back on stage. *J. Am. Soc. Nephrol.* 24, 161-163. 10.1681/ASN.2012121227 23334393

[DMM047464C28] Narita, K., Tsuruhara, T., Koenig, J. H. and Ikeda, K. (1989). Membrane pinch-off and reinsertion observed in living cells of *Drosophila*. *J. Cell Physiol.* 141, 383-391. 10.1002/jcp.1041410220 2808544

[DMM047464C29] Nguyen, N. N., Rana, A., Goldman, C., Moore, R., Tai, J., Hong, Y., Shen, J., Walker, D. W. and Hur, J. H. (2019). Proteasome β5 subunit overexpression improves proteostasis during aging and extends lifespan in *Drosophila* melanogaster. *Sci. Rep.* 9, 3170. 10.1038/s41598-019-39508-4 30816680 PMC6395709

[DMM047464C30] Ordonez, D. G., Lee, M. K. and Feany, M. B. (2018). α-synuclein induces mitochondrial dysfunction through spectrin and the actin cytoskeleton. *Neuron* 97, 108-124.e6. 10.1016/j.neuron.2017.11.036 29249285 PMC5755717

[DMM047464C31] Partridge, L. (2001). The insulin signaling pathway and aging in *Drosophila*. *ScientificWorldJournal* 1, 76. 10.1100/tsw.2001.138 PMC6083934 30147550

[DMM047464C32] Psathaki, O.-E., Dehnen, L., Hartley, P. S. and Paululat, A. (2018). *Drosophila* pericardial nephrocyte ultrastructure changes during ageing. *Mech. Ageing Dev.* 173, 9-20. 10.1016/j.mad.2018.04.006 29702130

[DMM047464C33] Pyo, J.-O., Yoo, S.-M., Ahn, H.-H., Nah, J., Hong, S.-H., Kam, T.-I., Jung, S. and Jung, Y.-K. (2013). Overexpression of Atg5 in mice activates autophagy and extends lifespan. *Nat. Commun.* 4, 2300. 10.1038/ncomms3300 23939249 PMC3753544

[DMM047464C34] Reenan, R. A. and Rogina, B. (2008). Acquired temperature-sensitive paralysis as a biomarker of declining neuronal function in aging *Drosophila*. *Aging Cell* 7, 179-186. 10.1111/j.1474-9726.2008.00368.x 18208580

[DMM047464C35] Revuelta, M. and Matheu, A. (2017). Autophagy in stem cell aging. *Aging Cell* 16, 912-915. 10.1111/acel.12655 28782921 PMC5595672

[DMM047464C36] Santra, M., Dill, K. A. and de Graff, A. M. R. (2019). Proteostasis collapse is a driver of cell aging and death. *Proc. Natl. Acad. Sci. USA* 116, 22173-22178. 10.1073/pnas.1906592116 31619571 PMC6825304

[DMM047464C37] Shirakabe, A., Ikeda, Y., Sciarretta, S., Zablocki, D. K. and Sadoshima, J. (2016). Aging and autophagy in the heart. *Circ. Res.* 118, 1563-1576. 10.1161/CIRCRESAHA.116.307474 27174950 PMC4869999

[DMM047464C38] Smith, L. K., He, Y., Park, J.-S., Bieri, G., Snethlage, C. E., Lin, K., Gontier, G., Wabl, R., Plambeck, K. E., Udeochu, J. et al. (2015). β2-microglobulin is a systemic pro-aging factor that impairs cognitive function and neurogenesis. *Nat. Med.* 21, 932-937. 10.1038/nm.3898 26147761 PMC4529371

[DMM047464C39] Sunderhaus, E. R. and Kretzschmar, D. (2016). Mass histology to quantify neurodegeneration in *Drosophila*. *J. Vis. Exp.* 54809. 0.3791/54809 10.3791/54809 PMC5226413 28060320

[DMM047464C40] Taylor, R. C. and Dillin, A. (2011). Aging as an event of proteostasis collapse. *Cold Spring Harb. Perspect. Biol.* 3, a004440. 10.1101/cshperspect.a004440 21441594 PMC3101847

[DMM047464C41] Tsakiri, E. N., Sykiotis, G. P., Papassideri, I. S., Terpos, E., Dimopoulos, M. A., Gorgoulis, V. G., Bohmann, D. and Trougakos, I. P. (2013). Proteasome dysfunction in *Drosophila* signals to an Nrf2-dependent regulatory circuit aiming to restore proteostasis and prevent premature aging. *Aging Cell* 12, 802-813. 10.1111/acel.12111 23738891 PMC4096703

[DMM047464C42] Weavers, H., Prieto-Sánchez, S., Grawe, F., Garcia-López, A., Artero, R., Wilsch-Bräuninger, M., Ruiz-Gómez, M., Skaer, H. and Denholm, B. (2009). The insect nephrocyte is a podocyte-like cell with a filtration slit diaphragm. *Nature* 457, 322-326. 10.1038/nature07526 18971929 PMC2687078

[DMM047464C43] Wittmann, C. W., Wszolek, M. F., Shulman, J. M., Salvaterra, P. M., Lewis, J., Hutton, M. and Feany, M. B. (2001). Tauopathy in *Drosophila*: neurodegeneration without neurofibrillary tangles. *Science* 293, 711-714. 10.1126/science.1062382 11408621

[DMM047464C44] Wong, S. Q., Kumar, A. V., Mills, J. and Lapierre, L. R. (2020). Autophagy in aging and longevity. *Hum. Genet.* 139, 277-290. 10.1007/s00439-019-02031-7 31144030 PMC6884674

[DMM047464C45] Zhang, F., Zhao, Y., Chao, Y., Muir, K. and Han, Z. (2013a). Cubilin and amnionless mediate protein reabsorption in *Drosophila* nephrocytes. *J. Am. Soc. Nephrol.* 24, 209-216. 10.1681/ASN.2012080795 23264686 PMC3559489

[DMM047464C46] Zhang, F., Zhao, Y. and Han, Z. (2013b). An in vivo functional analysis system for renal gene discovery in *Drosophila* pericardial nephrocytes. *J. Am. Soc. Nephrol.* 24, 191-197. 10.1681/ASN.2012080769 23291470 PMC3559487

